# ADS Score as a Novel Predictor of Outcomes in Patients Who Underwent Percutaneous Coronary Intervention

**DOI:** 10.3389/fcvm.2021.720597

**Published:** 2021-12-13

**Authors:** Wen-Jing Zhang, Gang-Qiong Liu, Jia-Hong Shangguan, Xiao-Dan Zhu, Wei Wang, Qian-Qian Guo, Jian-Chao Zhang, Kai Wang, Zhi-Yu Liu, Feng-Hua Song, Lei Fan, Ling Li, Ying-Ying Zheng, Jin-Ying Zhang

**Affiliations:** ^1^Department of Cardiology, First Affiliated Hospital of Zhengzhou University, Zhengzhou, China; ^2^Key Laboratory of Cardiac Injury and Repair of Henan Province, Zhengzhou, China; ^3^Henan Medical Association, Zhengzhou, China

**Keywords:** ADS score, mortality, major adverse cardiovascular and cerebrovascular events, major adverse cardiovascular events, coronary artery disease, percutaneous coronary intervention

## Abstract

**Objectives:** A novel AFR– albumin-derived neutrophil to lymphocyte ratio (dNLR) score (ADS) were reported to associate with clinical outcome in various malignancies, However, the relation between the ADS score and outcomes in coronary artery disease (CAD) patients after percutaneous coronary intervention (PCI) has not been investigated.

**Methods:** Three thousand five hundred and sixty-one patients were divided into two groups according to ADS score: low group (ADS score <2; *n* = 2,682) and high group (ADS score ≥ 2; *n* = 879). Overall, there were 133 all-cause mortality (ACM) during the following up. The incidence of ACM in the low group is 2.7% (72/2,682) and high group is 6.9% (61/879). The ACM incidence was significantly higher in high group compared to that in the low group (*P* < 0.001). Cardiac mortality (CM) occurred in 82 patients: 44(1.6%) in the low group and 38 (4.3%) in the high group. There was significant difference in the CM incidence between the low group and high group (*P* < 0.001). Major adverse cardiac and cerebrovascular events (MACCE) occurred in 520 patients: 366 (13.6%) in the low group and 154 (17.5%) in the high group. There was significant difference in the MACCE incidence between the low group and high group (*P* = 0.005). Major adverse cardiac and events (MACE) occurred in 395 patients: 281(10.5%) in the low group and 114 (13.0%) in the high group. There was significant difference in the MACE incidence between the low group and high group (*P* = 0.041). The multivariate Cox proportional hazards model showed that ADS score was independently correlated with the ACM [adjusted HR = 2.031 (1.357–3.039), *P* = 0.001]; CM [adjusted HR = 1.883 (1.127–3.147), *P* = 0.016]; MACCE [adjusted HR = 1.352 (1.096–1.668), *P* = 0.005], and MACE [adjusted HR = 1.260 (0.987–1.608), *P* = 0.063].

**Conclusion:** The present study indicated that the ADS score was associated with long-term mortality, the MACCE, and the MACE in CAD patients underwent PCI.

## Introduction

Chronic inflammation is considered to play a significant role on the occurrence and development of coronary artery disease (CAD) (1–4). There are a series of biomarkers of systematic inflammation such as inflammation-related immune cell and acute-phase reactive protein (5, 6). The levels of these biomarkers can reflect the degree of chronic inflammation in CAD patients. Previously, several inflammatory biomarkers, such as neutrophil to lymphocyte ratio (NLR) (7, 8), derived neutrophil to lymphocyte ratio (dNLR), fibrinogen (Fib) (9–12), and albumin (Alb) (13, 14), have been reported to be associated with the progress and prognosis of CAD. Fib is one of the vital elements in the coagulation cascade, and hypercoagulation is commonly occurred in CAD patients (15). Meanwhile, hypoalbuminemia was reported to be associated with outcomes of CAD (13, 14). Previous studies also suggested the ratio of Fib to albumin (FAR) was an independent predictor of CAD after percutaneous coronary intervention (PCI) (11, 16).

Previously, Gao et al. (17) developed a novel score system named ADS score which was composed of FAR, Alb, and dNLR to predict the prognosis of esophageal squamous cell carcinoma. As described above, all FAR, dNLR, and Alb were associated with the outcomes of CAD patients who underwent PCI. However, the association of ADS with prognosis of CAD patients has not been investigated up to date. In the present study, we investigated the predictive value of ADS score for the outcomes of CAD patients who underwent PCI.

## Patients and Methods

### Study Population

**Figure 1:**
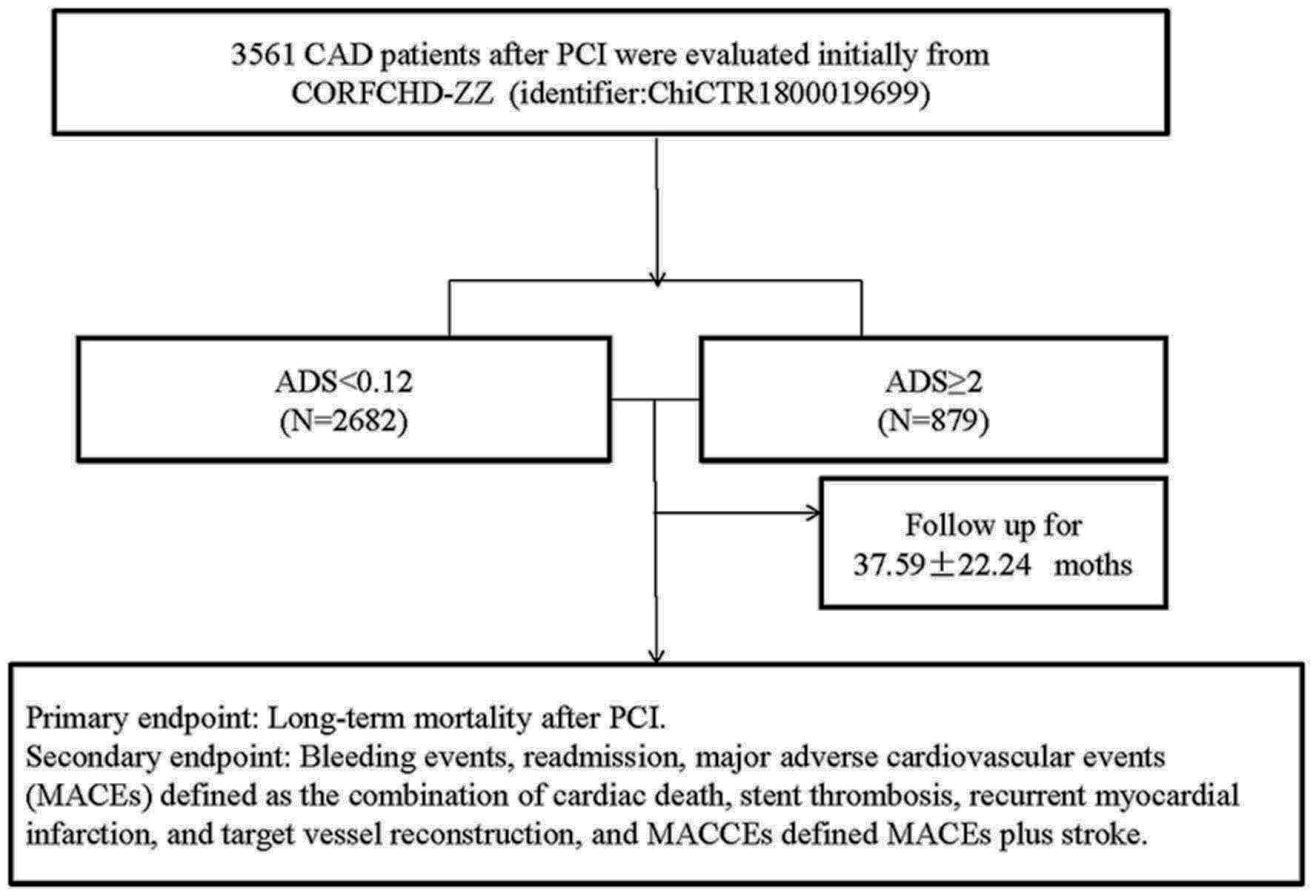
The flow chart of the study.

This study was a single-center retrospective cohort study that investigated the clinical outcomes and risk factors for patients with CAD after PCI (CORFCHD-ZZ, identifier: ChiCTR1800019699). In this study, 3,561 CAD patients who underwent PCI were enrolled and their clinical, angiographic, short-term, and long-term outcome data were collected. All the patients were from the First Affiliated Hospital of Zhengzhou University from 2013 to 2017. The selected criterion for CAD was at least one coronary artery diameter stenosis ≥70%, as confirmed on coronary angiography. An experienced cardiologist performed PCI. We divided these patients into two groups according to ADS score: lower group: ADS <2 (*n* = 2,682) and higher group: ADS ≥2 (*n* = 879). A flow chart of inclusion of the patients was shown in Figure 1.

### ADS Score Development

ADS score was established according to the previous study (17). Complete blood cell counts, such as total white blood cells, hemoglobin, neutrophils, platelets, lymphocytes, and monocytes were measured with an auto analyzer. Serum albumin levels were measured using a Roche Diagnostics Cobas 8000 c502 analyzer (Roche Holding AG, Basel, Switzerland). The normal range was 35–52 g/L. The calculation of AFR and dNLR was conducted according to the previous methods (11, 18). Levels of Alb, AFR, and dNLR which were higher or lower than the cut-off values were considered as 0 and 1 point, respectively. The total points with <2 and ≥2 were defined as low and high ADS score, respectively.

### Data Collection

The general demographic data, such as smoking, drinking, past medical history, cardiovascular risk factors, and laboratory-related tests were collected. All laboratory-related tests were performed on the second day after admission after fasting for at least 12 h. Serum concentrations of parameters representing liver function, kidney function, and blood lipid profiles were measured using the equipment for chemical analysis (Dimension AR/AVL Clinical Chemistry System, Newark, NJ, USA) employed by the Clinical Laboratory Department of the First Affiliated Hospital of Zhengzhou University.

### Definitions

In the present study, we defined hypertension as blood pressure ≥140/90 mmHg at three different times on the same day or treatment with antihypertensive drugs. The diagnostic criteria for diabetes was a clear history of diabetes, the use of hypoglycemic agents, fasting blood glucose ≥7.1 mmol/L, or 2 h post-load glucose ≥11.1 mmol/L. Persons reporting regular tobacco use in the previous 6 months were considered current smokers. Persons who were ingesting alcohol in the last 6 months were considered alcohol users. All the definitions were established according to the previous study (19).

Coronary angiography, interventional therapy, and post-operative reports were performed by experienced coronary intervention specialists. Patients with CAD who underwent PCI received a loading dose of DAPT (300 mg of aspirin and 300 mg of clopidogrel), and intravenous heparin anticoagulation was routinely used before PCI.

### Endpoints

The primary endpoint of the study was long-term mortality, including all-cause mortality (ACM), defined as death caused by any reason and cardiac mortality (CM), death due to coronary heart disease, cardiogenic shock, or sudden death. A combination of cardiac death, recurrent myocardial infarction, and target vessel reconstruction was defined major adverse cardiac events (MACEs) which was one of the secondary endpoints. The major adverse cardiac and cerebrovascular events (MACCEs), which were defined as a combination of cardiac death, recurrent myocardial infarction, target vessel reconstruction, and stroke, as described previously (19).

### Follow-Up

The follow-up was performed by specially trained professional staff. All patients were followed *via* outpatient, inpatient, and telephone follow-up and with questionnaire surveys. The mean follow-up time was 37.59 ± 22.24 months.

### Statistical Analysis

The SPSS 22.0 for Windows statistical software (SPSS Inc., Chicago, IL, USA) was used to perform the statistical analysis. The patients were divided into two groups according to ADS score. Continuous variables were expressed as the means ± standard deviation. The categorical variables were expressed as a percentage. One-way ANOVA was used to evaluate differences between normally distributed numerical variables, and non-normally distributed numerical variables were analyzed using the Mann–Whitney *U*-test. The Chi-squared test was used to compare the categorical variables. Kaplan–Meier analysis was utilized to analyze the cumulative incidence of long-term prognosis. The log-rank test was used to compare between groups. A multivariate Cox model was used to adjust the potential confounders. *P* < 0.05 was considered statistically significant.

## Results

### Baseline Data and Procedural Characteristics

**Figure 2:**
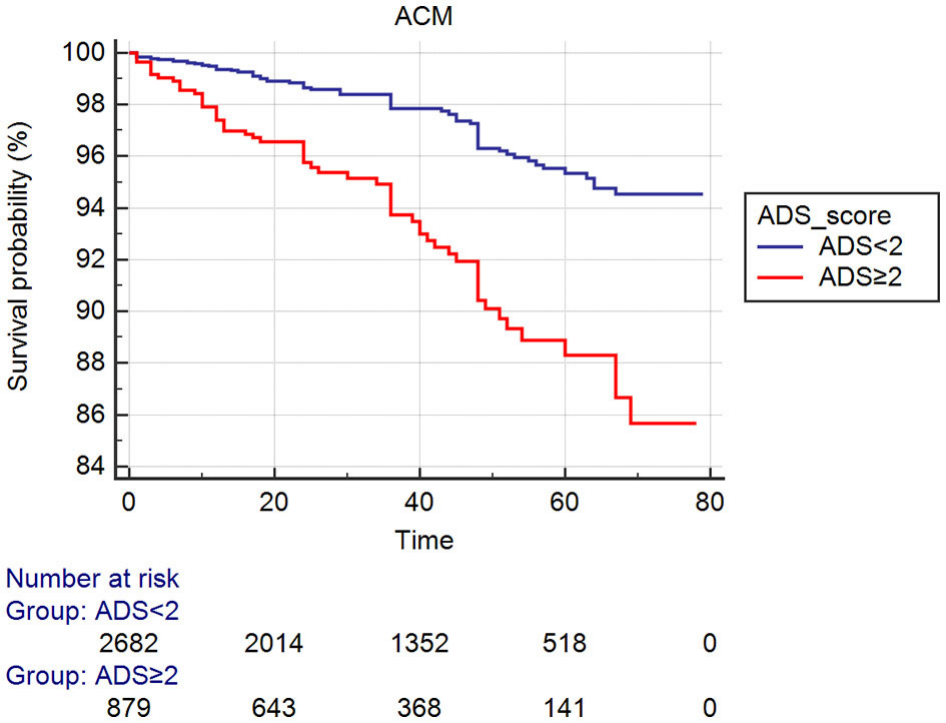
Cumulative Kaplan–Meier estimates of the time to the first adjudicated occurrence of primary endpoints (ACM).

**Figure 3:**
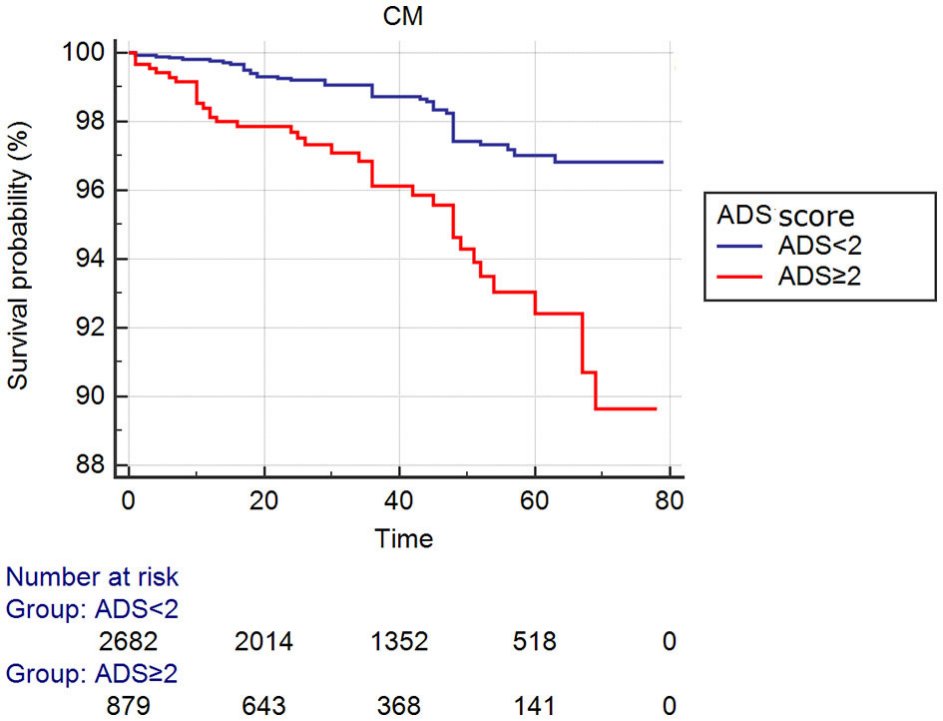
Cumulative Kaplan–Meier estimates of the time to the first adjudicated occurrence of primary endpoints (CM).

In the present study, we enrolled 3,561 patients who were divided into two groups according to ADS: lower group—ADS <2 (*n* = 2,682) and higher group—ADS ≥2 (*n* = 879). We found significant differences between the two groups in age, family history, Cr, GLU, TG, TC, HDL-C, medication of CCB, and statins (all *Ps* < 0.05). However, sex, male, family history, smoking, alcohol drinking, diabetes, hypertension, LDL-C, treatment of β-blockers, ACEI or ARB between groups were not significantly different (all *Ps* ≥ 0.05, Table 1, Figures 2, 3).

**Table 1:** Characteristics of participants of the two groups.

**Variables**	**ADS <2 (*n* = 2,682)**	**ADS ≥ 2 (*n* = 879)**	**χ** **^2^ or *t***	***P-*value**
Age, years	62.5 ± 10.5	65.5 ± 10.6	−7.246	**<0.001**
Sex, Male, *n* (%)	1,833 (68.3)	628 (71.4)	2.981	0.084
Family history, *n* (%)	528 (19.8)	138 (16.0)	6.307	**0.012**
Smoking, *n* (%)	805 (30.0)	278 (31.6)	0.813	0.367
Alcohol drinking, *n* (%)	427 (15.9)	150 (17.1)	0.638	0.424
Diabetes, *n* (%)	617 (23.0)	221 (25.1)	1.680	0.195
Hypertension, *n* (%)	1,485 (55.4)	488 (55.5)	0.006	0.939
Cr, umol/L	70.4 ± 29.2	79.7 ± 61.2	−5.886	**<0.001**
GLU, mmol/L	5.59 ± 2.26	6.07 ± 2.87	−4.779	**<0.001**
TG, mmol/L	3.43 ± 3.07	3.14 ± 1.73	2.444	**0.015**
TC, mmol/L	2.38 ± 1.52	2.20 ± 1.45	2.792	**0.005**
HDL-C, mmol/L	1.07 ± 0.43	0.98 ± 0.26	5.305	**<0.001**
LDL-C, mmol/L	2.54 ± 0.07	2.70 ± 0.33	−0.719	0.472
CCB, *n* (%)	485 (18.1)	133 (15.1)	4.024	**0.045**
β-blockers, *n* (%)	1,352 (50.4)	443 (50.4)	0.000	0.995
ACEI or ARB, *n* (%)	748 (27.9)	260 (29.6)	0.931	0.335
Statins, *n* (%)	2,175 (81.1)	652 (74.2)	19.379	**<0.001**

*Cr, Creatinine; GLU, Glucose; TG, Triglyceride; TC, Total cholesterol; HDL-C, High density lipoprotein cholesterol; LDL-C, Low density lipoprotein cholesterol; CCB, Calcium channel blocker; ACEI, Angiotensin-converting enzyme inhibitor; ARB, Angiotensin receptor blocker. The boldfaced P-values are statistically different*.

### Clinical Outcomes

As shown in Table 2, 133 patients developed ACM, such as 72 (2.7%) patients in the lower group and 61 (6.9%) patients in the higher group. The incidence of ACM increased gradually with the increase in ADS value, and the ACM incidence of the higher group was significantly increased than the lower group (HR = 2.852; 95% CI: 2.027–4.012, *P* < 0.001). We also found that the incidence of CM (HR = 2.920; 95% CI: 1.891–4.509, *P* < 0.001), MACCEs (HR = 1.427; 95% CI: 1.182–1.723, *P* < 0.001) or MACE (HR = 1.362; 95% CI: 1.095–1.693, *P* = 0.007) in the higher group was significantly greater compared to that in the lower group. These differences remained significant after multivariable COX regression analysis ACM [HR = 2.031 (95% CI: 1.357–3.039), *P* = 0.001], CM [HR = 1.883 (95% CI: 1.127–3.147), *P* = 0.016], and MACCEs [HR = 1.352 (95% CI: 1.096–1.668), *P* = 0.005]. However, the incidence of MACE was not significantly different between groups in multivariable analysis (HR = 1.260, 95% CI: 0.987–1.608, *P* = 0.063, Table 3).

**Table 2:** Outcomes comparison between groups.

**Outcomes**	**ADS <2 (*n* = 2,682)**	**ADS ≥ 2 (*n* = 879)**	***X* ^2^**	***P*-value**
ACM, *n* (%)	72 (2.7)	61 (6.9)	33.33	**<0.001**
CM, *n* (%)	44 (1.6)	38 (4.3)	21.18	**<0.001**
MACCEs, *n* (%)	366 (13.6)	154 (17.5)	7.965	**0.005**
MACEs, *n* (%)	281 (10.5)	114 (13.0)	4.169	**0.041**

*ACM, All-cause mortality; CM, Cardiac mortality; MACCEs, Major adverse cardiovascular and cerebrovascular events. MACEs, Major adverse cardiovascular events. The boldfaced P-values are statistically different*.

**Table 3:** Univariate and multivariate COX regression analysis results.

**Outcomes**	**HR (95%CI)**	***P*-value**	**Adjusted HR (95%CI)^**a**^**	***P*-value**
ACM	2.852 (2.027–4.012)	**<0.001**	2.031 (1.357–3.039)	**0.001**
CM	2.920 (1.891–4.509)	**<0.001**	1.883 (1.127–3.147)	**0.016**
MACCEs	1.427 (1.182–1.723)	**<0.001**	1.352 (1.096–1.668)	**0.005**
MACEs	1.362 (1.095–1.693)	**0.007**	1.260 (0.987–1.608)	0.063

*ACM, All-cause mortality; CM, Cardiac mortality; MACCEs, Major adverse cardiovascular and cerebrovascular events. MACEs, Major adverse cardiovascular events. The boldfaced P-values are statistically different.*

a *Adjusted for age, gender, family history of CAD, hypertension, diabetes, smoking, alcohol drinking, Cr, TC, HDL-C, LDL-C, and TG*.

## Discussion

Numerous biomarkers and scoring systems are used for prognostic evaluations of patients with CAD. However, some systems are relatively expensive or difficult to apply in clinical practice. The present study is the first to demonstrate the association of elevated ADS scores with an increased risk of adverse outcomes in CAD patients who underwent PCI.

Recent studies reported the association of inflammatory biomarkers with clinical outcomes of CAD (1–4). In our study, we comprehensively investigated the ADS score established with inflammatory related cells and ratios of them to predict the clinical outcomes of CAD. We found that ADS score was an efficacy predictor of CAD patients who underwent PCI. ADS score was established with Alb, dNLR, and AFR. Albumin is a major component of serum proteins. It contributes up to 80% of the total colloid osmotic pressure, transports drugs and endogenous compounds, acts as an antioxidant, and maintains microvascular integrity (20). The serum concentration of Albumin decreases in acute and chronic inflammatory states. Decreased serum albumin is associated with decreased fibrinolysis and enhanced platelet aggregation (21). A high ratio of fibrinogen to albumin accelerates erythrocyte aggregation in capillaries, which is also a cause of the occurrence of thrombotic disease (22). In addition, low albumin levels can facilitate hypercoagulability resulting in thrombotic events (23). Neutrophilia reflects the inflammation and the lymphocyte reflects the body's stress response; therefore, the dNLR represents a balance between these two paths (24, 25). The high dNLR represented an increased inflammatory response and the body's stress response. Previous study suggested that the NLR is an independent predictor of long-term cardiovascular outcome after elective PCI (26). These studies and findings provide strong evidence to support our current research.

Several limitations should be mentioned in the present study. First, there may be some unknown confounding factors, which affect the outcomes since this is a single-center observational study design. Second, the ADS score was constructed using the baseline parameters and its dynamic changes were not observed. Third, the mechanism of action between these parameters requires further study.

## Conclusion

The present study demonstrated that ADS was an effective and independent factor for predicting prognosis of CAD patients who underwent PCI.

## Data Availability Statement

The datasets presented in this article are not readily available due to confidentiality policies. Requests to access the datasets should be directed to Ying-Ying Zheng, zhengying527@163.com.

## Ethics Statement

The studies involving human participants were reviewed and approved by the Ethics Committee of the First Affiliated Hospital of Zhengzhou University. Written informed consent was not required for this study, in accordance with the local legislation and institutional requirements.

## Author Contributions

W-JZ, G-QL, J-HS, and X-DZ made substantial contributions to the study conception, design, and to the drafting and critical revision of the manuscript for important intellectual content. WW, Q-QG, J-CZ, Z-YL, F-HS, LF, and KW made substantial contributions to the study conception, design, and to the critical revision of the manuscript for important intellectual content. LL, J-YZ and Y-YZ made substantial contributions to the study conception, design, drafting, and critical revision of the manuscript for important intellectual content, such as study supervision. All authors contributed to the article and approved the submitted version.

## Funding

This work was funded by National Natural Science Foundation of China (Nos. 82000238 and 81870328) and Henan Medical Science and Technology Joint Building Program (No.2018020002), Henan Thousand Talents Program (No. ZYQR201912131).

## Conflict of Interest

The authors declare that the research was conducted in the absence of any commercial or financial relationships that could be construed as a potential conflict of interest.

## Publisher's Note

All claims expressed in this article are solely those of the authors and do not necessarily represent those of their affiliated organizations, or those of the publisher, the editors and the reviewers. Any product that may be evaluated in this article, or claim that may be made by its manufacturer, is not guaranteed or endorsed by the publisher.

## Abbreviations

CAD: coronary artery disease
ACM: all-cause mortality
MACE: major adverse cardiovascular events
CM: cardiac mortality
MACCEs: major adverse cardiovascular and cerebrovascular events
PCI: percutaneous coronary intervention.
